# Antileukemic effect of venetoclax and hypomethylating agents via caspase-3/GSDME-mediated pyroptosis

**DOI:** 10.1186/s12967-023-04481-0

**Published:** 2023-09-07

**Authors:** Fanghua Ye, Wen Zhang, Chenying Fan, Jiajia Dong, Min Peng, Wenjun Deng, Hui Zhang, Liangchun Yang

**Affiliations:** Department of Pediatrics, Xiangya Hospital, Central South University, 87 Xiangya Road, Changsha, Hunan 410008 People’s Republic of China

## Abstract

**Background:**

The identifying of B-cell lymphoma 2 (Bcl-2) as a therapeutic target has led to a paradigm shift in acute myeloid leukemia (AML) treatment. Pyroptosis is a novel antitumor therapeutic mechanism due to its cytotoxic and immunogenic effects. The combination of venetoclax and hypomethylating agents (HMAs) has been shown to lead to durable responses and significantly improve prognosis in patients with AML. However, our understanding of the mechanisms underlying this combinatorial activity is evolving.

**Methods:**

We investigated whether the Bcl-2 inhibitor venetoclax induces AML cell pyroptosis and identified pyroptosis effector proteins. Via using western blotting, immunoprecipitation, RNA interference, CCK8 assays, and LDH assays, we explored the mechanism underlying the pyroptotic effect. The relationship between the expression of the pyroptosis effector protein GSDME and AML prognosis was investigated. The effect of GSDME demethylation combined with venetoclax treatment on pyroptosis was investigated and confirmed in mouse models and clinical samples.

**Results:**

Venetoclax induces pyroptosis that is mediated by caspase-3-dependent GSDME cleavage. Mechanistically, venetoclax upregulates caspase-3 and GSDME cleavage by activating the intrinsic apoptotic pathway. GSDME is downregulated in AML by promoter methylation, and low GSDME expression is significantly associated with poor prognosis, based on public databases and patient sample analysis. In vivo and in vitro experiments showed that GSDME overexpression or HMAs-mediated restoration of GSDME expression significantly increased venetoclax-induced pyroptosis in AML.

**Conclusion:**

GSDME-mediated pyroptosis may be a novel aspect of the antileukemic effect of Bcl-2 inhibitors. This finding offers new insights into potential biomarkers and therapeutic strategies, identifying an important mechanism explaining the clinical activity of venetoclax and HMAs in AML.

**Supplementary Information:**

The online version contains supplementary material available at 10.1186/s12967-023-04481-0.

## Background

Acute myeloid leukemia (AML) is a heterogeneous malignancy characterized by clonal dysregulation of hematopoietic progenitors [1]. The “7 + 3” chemotherapy regimen (7 consecutive days of cytarabine followed by 3 days of anthracyclines) is the main treatment option for AML patients [2]. However, only approximately 60% of patients respond to this standard chemotherapy [3]. Patients have a poor prognosis due to severe side effects and acquired drug resistance [4]. Overexpression of B-cell lymphoma 2 (Bcl-2) is a clinical feature of human hematologic malignancies that exacerbates the malignant state and drives the apoptosis resistance phenotype [5, 6]. The identification of Bcl-2 as a therapeutic target ushered in a paradigm shift in the treatment of AML [7]. However, our understanding of the mechanisms of action of Bcl-2 inhibitors is still evolving.

Pyroptosis is a gasdermin (GSDM) protein-dependent mode of programmed cell death (PCD) characterized by membrane rupture via pores formed by the GSDM N-terminal protein, which is generated through caspase cleavage [8]. Induction of pyroptosis has emerged as a new therapeutic strategy for a variety of malignancies due to its dual cytotoxic and immunogenic effects [9–11]. Pyroptosis, characterized by swelling and rupture of cell membranes and leakage of cell contents such as lactate dehydrogenase (LDH), is mediated by several members of the GSDM superfamily, which consists of gasdermin A/B/C/D (GSDMA/B/C/D), gasdermin E (GSDME, also called DFNA5), and DFNB59 (also called Pejvakin, PJVK) [8]. Venetoclax (Ven) has been shown to induce apoptosis and autophagy [12]. However, its role in pyroptosis, a novel type of cell death, has not been characterized.

DNA methylation has been identified as an important mechanism for accelerating disease progression and drug resistance in AML by participating in the induction of genomic instability to regulate the expression of key genes [13]. Hypomethylating agents (HMAs), such as 5-azacytidine (5-aza) and decitabine, exhibit activity in AML [14]. Upon cellular uptake, 5-aza is metabolized and incorporated into DNA and RNA, depleting DNA methyltransferases (DNMTs) to drive DNA hypomethylation or nonepigenetic effects such as apoptosis induction [15, 16]. Recently, the combination of Ven and HMAs was shown to lead to durable responses and significantly improve prognosis in patients with both newly diagnosed and relapsed/refractory AML (RR-AML) [17–20]. However, the mechanism underlying the combinational activity of Ven and HMAs is incompletely elucidated.

In this study, we investigated the antileukemic effect of Ven and HMAs on inducing AML cell pyroptosis in vitro and in vivo by measuring the levels of pyroptosis markers, improving the pharmacological effect of Ven and identifying important mechanisms and insights into the combined use of Ven and HMAs for AML treatment.

## Materials and methods

### Patient samples

Bone marrow (BM) samples were collected from healthy BM transplant donors and patients with AML diagnosed between 2019 and 2022 at the Xiangya Hospital Central South University. The disease state of AML patients was evaluated based on the 2016 World Health Organization (WHO) criteria. After isolation with Histopaque^®^-1077 (Sigma‒Aldrich, USA), bone marrow mononuclear cells (BMMNCs) were stored at − 80 °C for RNA extraction. BMMNCs from patients with a naïve cell rate of > 75% were used as primary AML cells for subsequent analysis. Myeloid sarcoma (MS) and adjacent tissues from three patients with extramedullary MS were obtained from the pathology department of Xiangya Hospital. The clinicopathological characteristics of the patients are shown in Additional file 1: Table S1. This study was approved by the Ethics Committee of Xiangya Hospital Central South University. All patients and donors provided written informed consent.

### Cell culture and reagents

The AML cell lines Molm-13, HL-60, THP-1, Kasumi-1, and MV4-11 were obtained from the American Type Culture Collection. Cells were cultured in RPMI 1640 medium supplemented with 10% fetal bovine serum (FBS) (Gibco) and 1% penicillin/streptomycin (Beyotime). All cells were grown at 37 °C in a 5% CO_2_ incubator.

Ven (Bcl-2 inhibitor, #HY-15531) was purchased from MedChemExpress (Shanghai, China). Z-DEVD-FMK (caspase-3 (CASP3) inhibitor, #S7312), Decitabine / Dacogen (Dac; #S1200), puromycin (#S7417), and N-acetylcysteine (NAC, #S1623) were supplied by Selleck (Houston, USA). A Bcl-2 associated X (Bax) channel blocker (Bcb; #A4459) was purchased from APExBIO (Houston, USA).

### Cell viability assay

Cell viability was evaluated with a Cell Counting Kit-8 (CCK-8; APE x BIO Technology) according to the manufacturer’s instructions. The data are presented as the percent (%) of viable cells relative to that in the control group.

### Lactate dehydrogenase (LDH) release assay

Cytotoxicity was evaluated using an LDH cytotoxicity assay kit (Beyotime) according to the manufacturer's instructions. The amount of LDH released from the damaged cell membrane, expressed as a fold change relative to the control, was used to quantify the integrity of the cell membrane.

### Plasmids and lentiviral transduction

The knockdown cell lines were generated using lentivirus-packaged GSDME short hairpin RNA (shRNA) (GeneChem Inc., Shanghai, China). The GSDME shRNA oligonucleotide sequences are listed in Additional file 1: Table S2. The pCMV-GSDME plasmid was constructed by inserting GSDME cDNA into the GV492 expression vector. Forty-eight hours after infection with lentivirus, cells were incubated in complete RPMI 1640 medium containing puromycin to establish stably transduced cell lines.

### RNA extraction and reverse transcription–quantitative PCR (RT–qPCR)

TRIzol reagent (Invitrogen, USA) was used according to the manufacturer’s instructions, and cDNA synthesis was performed using PrimeScript™ RT Master Mix (Yeasen Biotech). A 7900 Real-Time PCR System (Applied Biosystems, Foster City, CA) with Hieff^®^ qPCR SYBR Green Master Mix (Low Rox Plus, Yeasen Biotech) was used for qPCR, and target mRNA expression levels were normalized to that of the housekeeping gene β-actin. Relative gene expression levels were calculated by the 2^−ΔΔCt^ method. The primers used for RT-qPCR are listed in Additional file 1: Table S3.

### Western blot analysis and immunoprecipitation (IP)

Cells were lysed in RIPA buffer containing a phosphatase inhibitor and protease inhibitor cocktail. Protein concentrations in the extracts were determined using a bicinchoninic acid (BCA) assay (Beyotime). Sodium dodecyl sulfate–polyacrylamide gel electrophoresis (SDS‒PAGE) was used to separate extracted proteins (30 µg/lane), which were then electrotransferred to polyvinylidene fluoride (PVDF) membranes. After blocking with 5% nonfat milk for 2 h at room temperature, the membranes were incubated with primary antibodies for 24 h at 4 °C and were then incubated with secondary antibodies. Mitochondria were isolated by using a Cell Mitochondria Isolation Kit (Beyotime). For immunoprecipitation, equal amounts of protein were incubated with primary antibodies at 4 °C. Protein A/G agarose beads (Beyotime) were used to isolate immune complexes which were then washed 5 times in ice-cold PBS and finally analyzed by western blotting. Information on the antibodies is listed in Additional file 1: Table S4.

### Hoechst 33342/propidium iodide (PI) staining

Cell lines were treated with Ven as appropriate and were then stained with Hoechst 33342 and PI (Solarbio) for 30 min in the dark. Staining was visualized by fluorescence microscopy (Nikon, A1 + , Tokyo, Japan).

### Mitochondrial membrane potential (MMP) assay

Cells were treated with Ven before measurement of the MMP by using a Mitochondrial Membrane Potential Assay Kit with TMRE (Beyotime) in accordance with the manufacturer’s instructions. The MMP was analyzed by fluorescence microscopy or fluorescence photometry (F4500, Hitachi, Tokyo, Japan).

### Reactive oxygen species (ROS) measurement

Intracellular changes in ROS levels were determined by quantifying the oxidative conversion of the cell-permeable dye 2′,7′-dichlorofluorescein diacetate (DCFH-DA) (Beyotime) to fluorescent dichlorofluorescein (DCF) on a fluorescence photometer (F4500, Hitachi, Tokyo, Japan).

### Bisulfite sequencing PCR (BSP) and methylation-specific PCR (MSP)

Genomic DNA was extracted using a Genomic DNA Kit (Tiangen Biotech) for analysis of GSDME promoter methylation by BSP and MSP. Genomic DNA was subjected to bisulfite treatment using a DNA Methylation Gold Kit (ZYMO). Bisulfite-treated DNA was amplified with primers specific for either methylated or unmethylated DNA. The sequences of the BSP primers, methylated DNA-specific (M) primers and unmethylated DNA-specific (U) primers used for GSDME amplification are listed in Additional file 1: Table S5.

### Analysis of patient

Patient data and gene expression datasets were obtained from online database, including Gene Expression Profiling Interactive Analysis (GEPIA; http://gepia2.cancer-pku.cn), Cancer Cell Line Encyclopedia (CCLE; https://sites.broadinstitute.org/ccle), Human Protein Atlas (HPA; https://www.proteinatlas.org/), R2: microarray analysis and visualization platform (http://hgserver1.amc.nl/cgibin/r2/main.cgi), Kaplan‒Meier Plotter (https://kmplot.com/analysis/) and DiseaseMeth version 2.0 (http://bio-bigdata.hrbmu.edu.cn/diseasemeth/index.html). The figures and *P* values related to these analyses were downloaded.

### Subcutaneous AML xenograft model in mice

HL-60 cells (5 × 10^6^ cells/mouse) were resuspended in 100 μL of matrigel (Corning) and subcutaneously injected into the right flanks of 5-week-old male NOD/SCID mice, purchased from the Laboratory Animal Center of Central South University. When the tumor volume was approximately 60 mm^3^, the mice were randomly divided into four groups (n = 5 mice per group): vehicle control, decitabine (Dac), Ven, and Ven + Dac (combination treatment). Leukemia-bearing mice were treated with 1 mg/kg decitabine via intraperitoneal injection once daily for five days, 100 mg/kg Ven via orally once daily for 14 days, 1 mg/kg decitabine once daily for five days followed by 100 mg/kg Ven once daily for 14 days, or vehicle control. The mice were weighed every 3 days. The tumor size was measured every 2 or 3 days using digital calipers. The tumour volume was calculated using the following equation: volume = 0.52 × L × W^2^. Ultimately, the mice were sacrificed, and the tumors were excised for subsequent assays. All procedures involving animals were reviewed and approved by the Animal Care and Welfare Committee of Central South University.

### Immunohistochemical staining (IHC)

Tumor tissues from mice and MS tissues and paired adjacent tissues from human patients were fixed with 10% formalin for 24 h. After dehydration and paraffin embedding, the specimens were sectioned at 5 μm using a microtome (Leica, Wetzlar, Germany) and mounted on glass slides. The protein levels of Ki-67, GSDME, and cleaved caspase-3 (Cl-CASP3) were measured with an IHC assay kit (Boster). The sources and dilutions of the primary antibodies used are listed in Additional file 1: Table S4. Antibody staining in tissue sections was observed at 40 × magnification. Staining intensities were quantified using IHC Profiler, a plugin in ImageJ software, to determine Histo scores (H-scores), which were directly proportional to the concentration of DAB [21].

### Terminal deoxynucleotidyl transferase dUTP nick end labeling (TUNEL) assay

A TUNEL assay (Beyotime) was performed on tumor tissues according to the manufacturer's instructions. After labeling, nuclei were counterstained with 4’,6- diamidino-2-phenylindole (DAPI). TUNEL-positive cells were photographed using a fluorescence microscope (Nikon, A1 + , Tokyo, Japan).

### Statistical analysis

GraphPad Prism 8.0 (GraphPad Software Inc., San Diego, CA, USA) was used for statistical analysis and graphing. Data are presented as the mean ± standard deviation (SD) of at least three independent experiments. Comparisons between the two groups were performed by Student's t test. One-way analysis of variance (ANOVA) was used for comparisons among multiple groups, and two-way ANOVA was used for comparisons among multiple groups with two independent variables. *P* < 0.05 was considered to indicate a statistically significant difference.

## Results

### Ven activates GSDME-mediated pyroptosis in AML cells

**Fig. 1:**
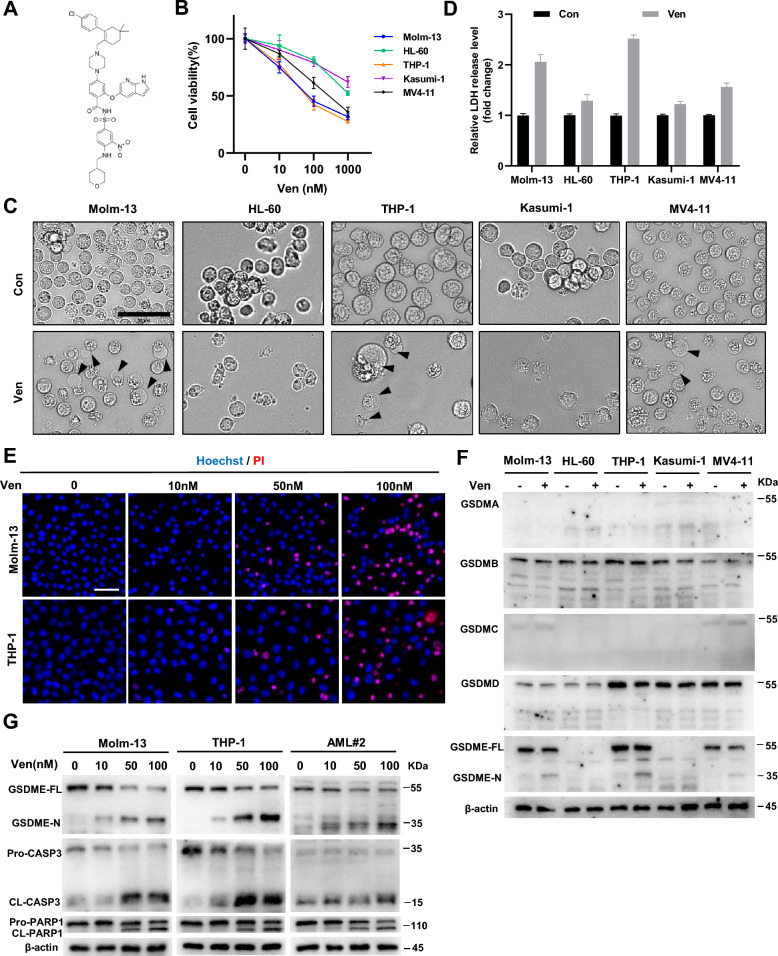
Ven activates GSDME-mediated pyroptosis in AML cells. **A** The chemical formula of Ven. **B** Viability of five AML cell lines treated with different concentrations of Ven for 24 h. **C** Micrographs of the five AML cell lines treated with Ven. The arrowheads indicate blebbed pyroptotic cells; scale bar: 50 μm. **D** Release of LDH into the culture supernatant was detected by an LDH assay kit. **E** Ven-treated Molm-3 and THP-1 cells were subjected to Hoechst/PI staining. Nuclei were stained blue with Hoechst, and red with PI. **F** AML cells were treated with Ven, and cell lysates were then subjected to western blot analysis to confirm GSDM protein expression and cleavage. **G** Protein levels of full-length (FL) and N-terminal of GSDME, pro-caspase-3 (Pro-CASP3) and cleaved caspase-3 (CL-CASP3), pro-PARP1 and cleaved PARP1 in Ven-treated Molm-13 cells, THP-1 cells, and primary cells from AML patients. β-actin was used as an internal control for western blotting

Ven (Fig. 1A), the first-in-class Bcl-2 antagonist, has shown promising benefits in AML [22]. The proliferation of five different AML cell lines was inhibited to varying degrees by treatment with Ven at concentrations ranging from 10 to 1000 nM for 24 h (Fig. 1B). Molm-13, THP-1 and MV4-11 cells but not HL-60 and Kasumi-1 cells showed blebbing of the cell membrane and nucleus pushed sideways, a typical early features of pyroptosis (Fig. 1C) [23]. Consistent with these morphological changes, increases in LDH levels were more pronounced in the culture medium of these cell lines (Fig. 1D). The rate of positive PI staining gradually increased with increasing concentrations of Ven (Fig. 1E), indicating that the plasma membrane of these cells was disrupted, a hallmark of pyroptosis. These findings suggest that Ven induces pyroptosis in AML cells. Pyroptosis depends on the cleavage and activation of GSDM proteins [24]. Thus, we next determined the expression profiles of GSDMs in AML cell lines. GSDME, but not GSDMA, GSDMB, GSDMC, or GSDMD was cleaved upon Ven treatment (Fig. 1F). Chemotherapeutics activate caspase-3 for cleavage of GSDME [23]. As expected, Ven activated caspase-3, poly ADP-ribose polymerase 1 (PARP1), and GSDME in a concentration-dependent manner in Molm-13 and THP-1 cells as well as in primary cells from AML patients (Fig. 1G). Thus, in addition to triggering caspase-3/PARP1-mediated apoptosis, Ven induces caspase-3/GSDME-dependent pyroptosis in AML cells.

### Caspase-3-mediated cleavage of GSDME is involved in Ven- induced pyroptosis in AML cells

**Fig. 2:**
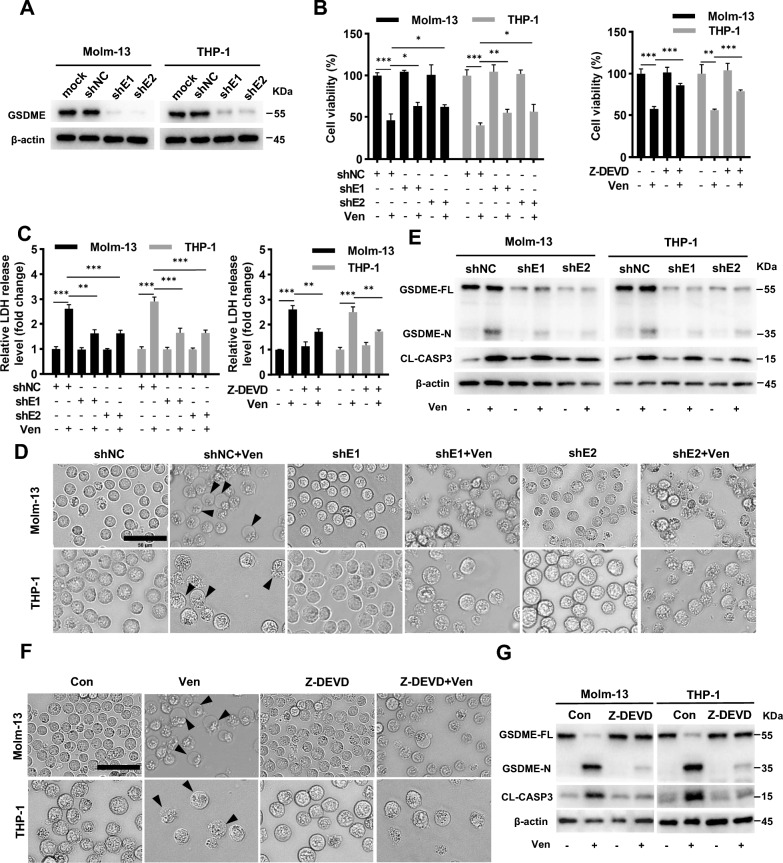
Caspase-3-mediated cleavage of GSDME is involved in Ven-induced pyroptosis in AML cells. **A** Validation of shRNA-mediated knockdown of GSDME in Molm-13 and THP-1 cells by Western blotting. Knockdown of GSDME or pretreatment with the caspase-3 inhibitor Z-DEVD-FMK (10 µM) for 12 h was followed by treatment with Ven (100 nM) for 24 h, **B** Cell viability was evaluated by a CCK8 assay, and **C** the release of LDH into the culture supernatant was detected by an LDH assay kit. (**D** and **F**) Micrographs. The black arrows indicate blebbed cell membranes; scale bar = 50 μm. (**E** and **G**) Western blotting of GSDME-N and CL-CASP3. β-actin was used as an internal control for western blotting. ^∗^*p* < 0.05, ^∗∗^*p* < 0.01, and.^∗∗∗^*p* < 0.001

The above data suggested that Ven-induced pyroptosis in AML cells was GSDME dependent. Here, to validate the role of GSDME,we stably knocked down GSDME in Molm-13 and THP-1 cells using lentiviral shRNAs (Fig. 2A). After knockdown of GSDME, Ven-induced proliferation inhibition (Fig. 2B), LDH release (Fig. 2C), cell membrane blebbing (Fig. 2D), and GSDME cleavage (Fig. 2E) were significantly attenuated. Similar inhibitory effects were observed when cells were pretreated with Ven and the caspase-3 inhibitor Z-DEVD-FMK, a specific, irreversible inhibitor of caspase-3 that significantly inhibits caspase-3 activation, for 12 h before Ven treatment. After caspase-3 inhibitor treatment, Ven-induced proliferation inhibition (Fig. 2B), LDH release (Fig. 2C), cell membrane blebbing (Fig. 2F), and GSDME and CASP3 cleavage (Fig. 2G) were significantly suppressed. Regardless of apoptosis activity, inhibition of caspase-3 significantly suppressed Ven-induced LDH release and GSDME cleavage, suggesting that caspase-3-mediated cleavage of GSDME is required for Ven-induced pyroptosis.

### Ven activates caspase-3 -mediated cleavage of GSDME via the intrinsic apoptotic pathway

**Fig. 3:**
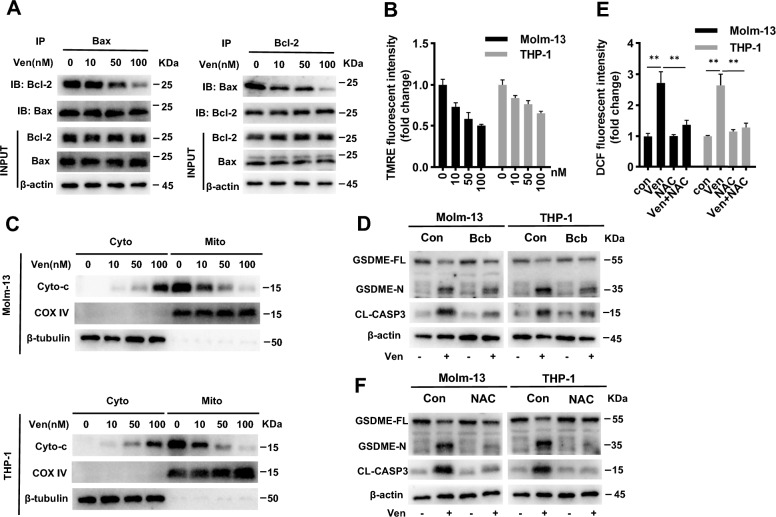
Ven activates caspase-3 -mediated cleavage of GSDME via the intrinsic apoptotic pathway. **A** Molm-3 and THP-1 cells were treated with Ven for 24 h, and the cell lysates were then prepared for immunoprecipitation followed by western blotting as indicated. **B** Molm-3/THP-1 cells were stained with TMRE after Ven treatment and the relative fluorescence intensity was measured. **C** Molm-13/THP-1 cells were treated with Ven, followed by isolation of the mitochondrial and cytosolic fractions and western blot analysis as indicated. **D** Cells were pretreated with Bcb (10 µM) for 12 h prior to treatment with Ven (100 nM) for 24 h, and GSDME-N and CL-CASP3 were assayed by western blotting. Molm-13/THP-1 cells were pretreated with or without NAC (20 μM) for 12 h and were then treated with Ven (100 nM) for 24 h, ROS levels were quantified by measuring the relative DCF fluorescence intensity (**E**), GSDME-N, and CL-CASP3 were analyzed by western blotng (**F**). β-actin was used as an internal control for western blotting. ^∗∗^*p* < 0.01

Ven is a Bcl-2 inhibitor that induces mitochondrial dysfunction [25]. Important indicators of decreased mitochondrial integrity are mitochondrial cytochrome C leakage and MMP loss [26, 27]. Therefore, we investigated the interaction between Bcl-2 and Bax in the presence of Ven. Reciprocal immunoprecipitation assays showed that Ven significantly reduced the Bcl-2/Bax interaction (Fig. 3A). TMRE is a red cationic fluorescent probe that can pass through cell membranes and accumulate in intact mitochondria, and its accumulation is reduced in depolarized or damaged mitochondria [28]. The TMRE fluorescence intensity was significantly decreased in Ven-treated AML cells compared to control cells (Fig. 3B). Consistent results were found by fluorescence microscopy (Additional file 1: Fig. S1). Furthermore, cytochrome C levels in mitochondria were decreased after Ven treatment, while cytoplasmic cytochrome C levels increased accordingly, consistent with the trends in caspase-3 activation and GSDME cleavage (Fig. 3C). Bcb, which inhibits cytochrome C release, suppressed Ven-induced caspase-3 activation and GSDME cleavage (Fig. 3D). Mitochondria are a major source of intracellular ROS [29]. ROS have been shown to induce cellular damage [30, 31]. Moreover, it is postulated that Ven may increase cellular ROS levels due to the close relationship between mitochondrial dysfunction and ROS production. Fluorescence analysis showed that ROS levels were increased after Ven treatment. However, NAC, a potent ROS scavenger, effectively antagonized this process (Fig. 3E). Moreover, NAC greatly attenuated the cleavage of caspase-3/GSDME (Fig. 3F). Taken together, these findings suggest that Ven triggers cytochrome C release and ROS generation by disrupting the MMP, ultimately leading to caspase-3/GSDME-mediated pyroptosis in AML cells.

### GSDME overexpression enhances Ven-induced pyroptosis in AML cells

**Fig. 4:**
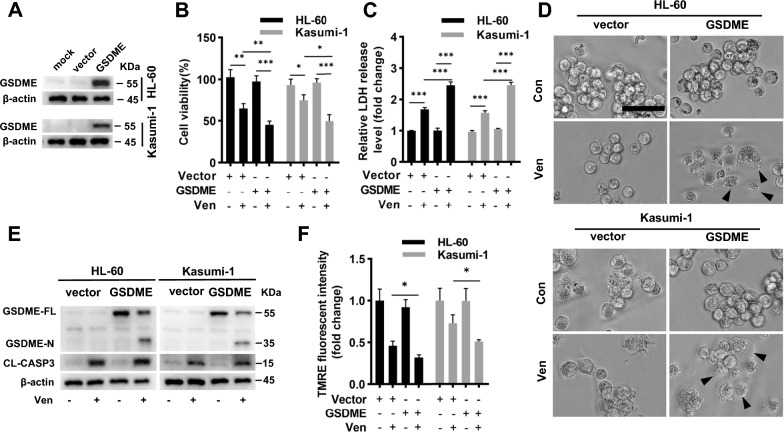
GSDME overexpression enhances Ven-induced pyroptosis in AML cells. **A** Validation of GSDME overexpression in Molm-13 and THP-1 cells by Western blotting. Cell viability assay (**B**), LDH release assay (**C**), micrographs (**D**), and quantification of GSDME and caspase-3 cleavage (**E**) in GSDME-overexpressing HL-60 and Kasumi-1 cells treated with Ven (100 nM) for 24 h. Scale bar = 50 μm. **F** HL-60/Kasumi-1 cells were stained with TMRE, and the relative fluorescence intensity was measured. β-actin was used as an internal control for western blotting. ^∗^*p* < 0.05, ^∗∗^*p* < 0.01, and.^∗∗∗^*p* < 0.001

HL-60 and Kasumi-1 are two typical AML cell lines lacking GSDME expression (Fig. 1F). We thus introduced GSDME in these two cell lines using lentiviral transduction. GSDME overexpression was confirmed by western blotting (Fig. 4A). Although overexpressing GSDME alone did not affect cell viability, it synergistically enhanced the Ven-induced inhibition of proliferation and release of LDH in these cells (Fig. 4B and C). In wild-type HL-60 and Kasumi-1 cells, Ven treatment did not result in morphological changes suggestive of pyroptosis, but such changes were observed when GSDME was introduced into these cells (Fig. 4D). Furthermore, consistent with the inhibition of proliferation, release of LDH, and morphological changes, GSDME cleavage was increased (Fig. 4E). TMRE staining showed that the MMP was more markedly reduced in GSDME-overexpressing HL-60 and Kasumi-1 cells after Ven treatment (Fig. 4F). This pattern may also explain why overexpression of GSDME increased cell death. Taken together, these data demonstrated the additive effect of GSDME overexpression on Ven-induced pyroptosis.

### GSDME expression is downregulated in AML, and GSDME downregulation is associated with poor prognosis

**Fig. 5:**
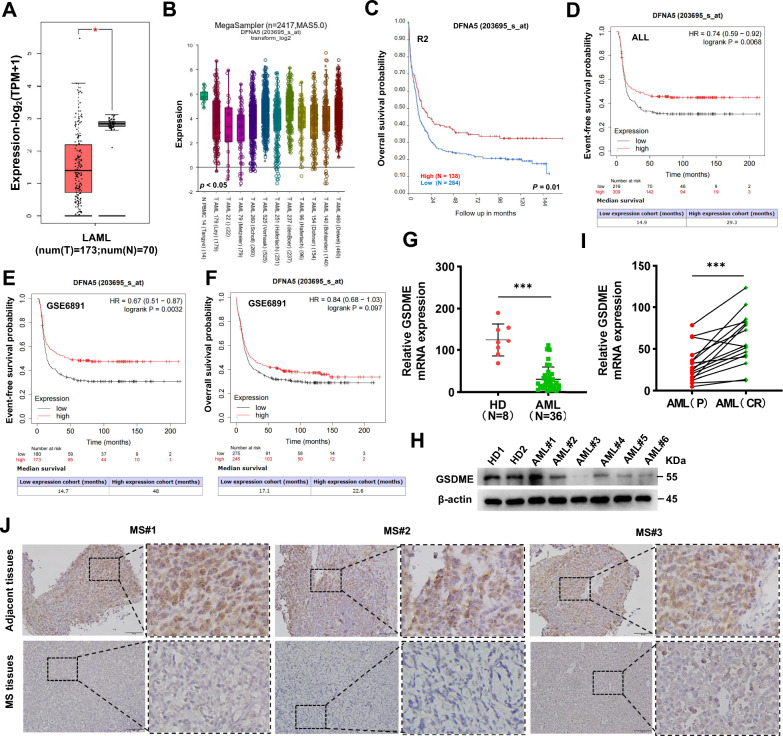
GSDME expression is downregulated in AML, and GSDME downregulation is was associated with poor prognosis. **A** The expression of GSDME in AML patients (n = 173) from TCGA and normal controls (n = 70) from the GTX database was compared by using (GEPIA) (*p* < 0.01). **B** The expression of GSDME (DFNA5) in AML cells was compared with that in normal PBMCs in the R2 databases. **C**-**F** Analysis of overall survival and event-free survival in leukemia patients with high DFNA5 expression versus leukemia patients with low-DFNA5 expression in the R2 and Kaplan‒Meier Plotter databases. **G** The expression level of GSDME mRNA in healthy donors (n = 8) and patients with de novo AML (n = 36) (mean ± SEM: 124.7 ± 13.69 vs. 30.72 ± 4.817, *p* < 0.001). **H** Western blot analysis of GSDME protein expression in primary AML blasts and the counterparts cells from HD. **I** The GSDME expression in AML patients with a complete response after chemotherapy was significantly higher than that at baseline (*p* = 0.004) (n = 16 paired groups). (J) IHC analysis of GSDME expression in MS tissues and adjacent tissues

By analyzing more than 30 different types of human cancer cell lines in the CCLE database, we found that GSDME was expressed at low levels in AML cell lines (Additional file 1: Fig. S2A). Low GSDME protein expression in AML cell lines was also reported in the HPA public database (Additional file 1: Fig. S2B). We further analyzed GSDME expression using the GEPIA database and found aberrant expression of GSDME in 33 human cancers (Additional file 1: Fig. S2C). Moreover, GSDME mRNA expression was significantly lower in tissues from 173 patients with newly diagnosed AML than in normal tissues from 70 patients in the Genotype–Tissue Expression (GTEx) database (Fig. 5A). The potential prognostic value of GSDME expression in patients with leukemia patients was investigated using the R2 and Kaplan‒Meier Plotter databases. The R2 genomic analysis indicated that the mRNA expression of GSDME was significantly downregulated in AML patient datasets compared to the normal leukocyte/control dataset (Fig. 5B). Additionally, reduced expression of GSDME was significantly associated with decreased event-free survival in leukemia patients, as determined by analysis with Kaplan‒Meier Plotter (Fig. 5C and D). Although there was no significant difference in overall survival, the median survival time was longer in patients with high GSDME expression than in those with low expression (Fig. 5F). Another cohort of 8 healthy donors (HD) and 36 patients with newly diagnosed AML was recruited from Xiangya Hospital. qRT‒PCR (Fig. 5G), and western blotting (Fig. 5H) were used to quantify the expression of GSDME. GSDME expression was lower in AML patients than in healthy donors and was higher after complete remission after chemotherapy than at diagnosis (Fig. 5I). Furthermore, we observed reduced GSDME expression in tissues of MS, an entity of AML, compared to adjacent tissues (Fig. 5J). In the 36 AML patients, however, no significant correlations were found between clinicopathologic parameters and GSDME expression levels (Additional file 1: Table S1). GSDME expression was positively correlated with the prognosis of AML patients and mediated pyroptosis in AML cells, suggesting that it may act as a suppressor of AML.

### GSDME demethylation enhances Ven-induced pyroptosis in AML cells

**Fig. 6:**
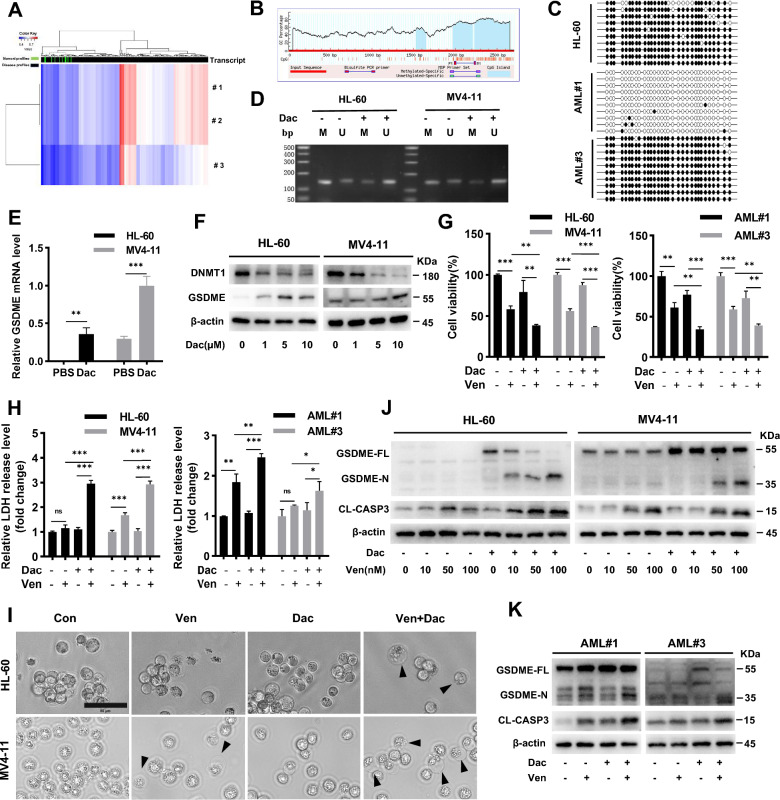
GSDME demethylation enhances Ven-induced pyroptosis in AML cells. **A** Heatmap of GSDME methylation in AML generated from methylation data of 3 transcripts from 281 samples in a 450 k array. The rows represent transcripts, and the columns represent samples (green: normal profiles, black: disease profiles). **B** MethPrimer software was used to identify potential methylation sites in GSDME CpG islands. **C** The methylation status of the GSDME promoter in AML cell lines and primary cells was verified by BSP sequencing. The black dots represent methylated cytosine residues in the CpG islands, and the white dots represent unmethylated CpG dinucleotides. **D** MSP profiles of the GSDME promoter methylation status in HL-60 and MV4-11 cells before and after pretreatment with decitabine for 72 h; M, methylated; U, unmethylated. After 72 h of decitabine pretreatment, GSDME mRNA expression was quantified by qRT‒PCR (**E**) and western blotting (**F**). After Ven treatment with/without decitabine pretreatment, cell viability (**G**), LDH release (**H**), microscopy (**I**), and protein cleavage of GSDME and caspase-3 (**J**-**K**) were examined in HL-60/MV4-11 cells and primary AML cells. Scale bar = 50 μm. β-actin was used as an internal control for western blotting. ^∗^*p* < 0.05, ^∗∗^*p* < 0.01, and.^∗∗∗^*p* < 0.001, and *p* > 0.05 not significant (ns)

GSDME expression is silencedn a variety of tumors due to promoter methylation [32, 33]. DNA methyltransferase-1 (DNMT1) is responsible for maintaining established methylation and plays a potential role in promoting the progression of AML and drug resistance [34]. AML patients have higher GSDME promoter methylation levels than healthy donors, according to the methylation heatmap in DiseaseMeth version 2.0 (Fig. 6A and Additional file 1: Table S6). Methylation sites in the GSDME CpG islands were identified using the MethPrimer software (Fig. 6B). BSP analysis of the GSDME gene promoter was performed in HL-60 and primary AML cells (Fig. 6C). Decitabine treatment of HL-60 and MV4-11 cells resulted in GSDME demethylation (Fig. 6D), restored GSDME mRNA transcription (Fig. 6E), reduced the expression level of DNMT1, and increased the GSDME protein expression (Fig. 6F). The effects on cell viability (Fig. 6G), LDH release (Fig. 6H), cell membrane blebbing (Fig. 6I, Additional file 1: Fig. S3), and GSDME/caspase-3 cleavage (Fig. 6J, K) were enhanced after pretreatment with decitabine compared to treatment with Ven alone in both AML cell lines and patient-derived primary AML cells. In addition, compared with wild-type HL-60 and MV4-11 cells, the combination treatment resulted in decreased LDH release in GSDME knockdown HL-60 and MV4-11 cells (Additional file 1: Fig. S4), further demonstrating the important role of GSDME expression in the combinatorial effect. These results indicated that demethylation of GSDME facilitated Ven-induced proliferation inhibition and pyroptosis in AML cells.

### GSDME demethylation promotes Ven-induced pyroptosis in AML cells in vivo.

**Fig. 7:**
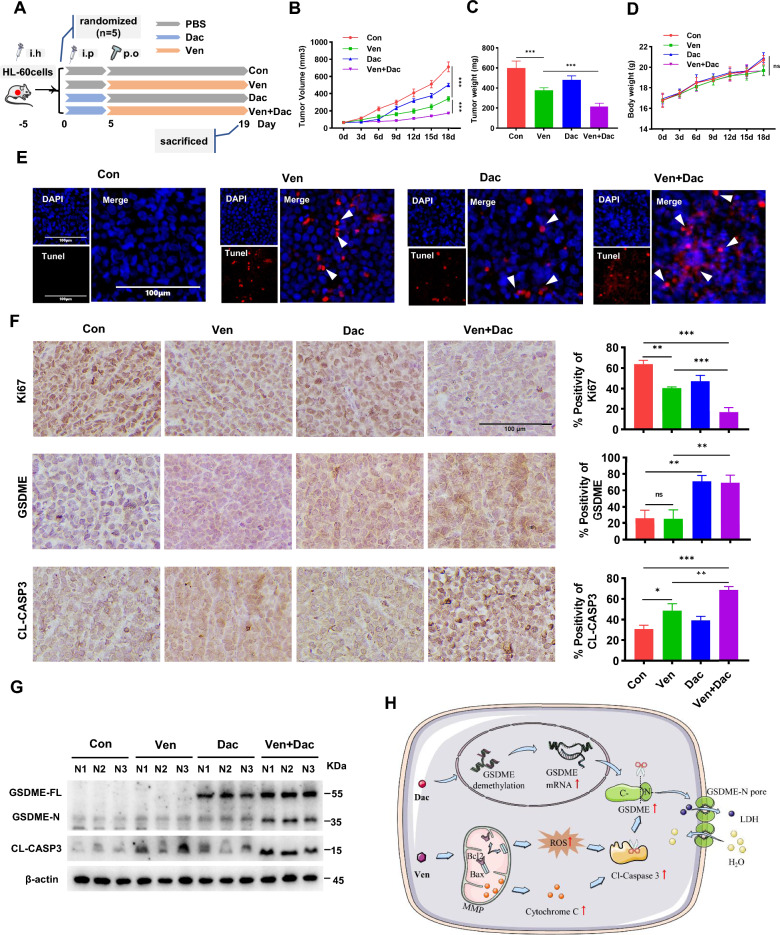
GSDME demethylation promotes Ven-induced pyroptosis in AML cells in vivo. **A** Schematic of treatment regimens. **B** Tumor volumes during the treatment period (n = 5). **C** Tumor weights at the end of the experiment (n = 5). **D** Average body weight of mice in each group during the treatment period (n = 5). **E** TUNEL assay of xenograft tumor sections in each group. The arrow indicates TUNEL positivity. **F** Images of IHC staining for Ki67, Cl-casp3, and GSDME in tumor tissues; scale bar: 100 µm. The bar chart shows the quantitative results. **G** Western blot analysis of GSDME and caspase-3 protein cleavage in tumor tissues (n = 3). ^∗^*p* < 0.05; ^∗∗^*p* < 0.01; and ^∗∗∗^*p* < 0.001; and *p* > 0.05 not significant (ns). **H** Schematic representation of the mechanisms underlying the induction of pyroptosis in AML cells by a combined treatment with decitabine and Ven via caspase-3/GSDME activation

AML xenografts were established by subcutaneous transplantation of HL-60 cells into NOD/SCID mice (Fig. 7A). Compared with the control group, the Dac group, Ven group, and the combination treatment group exhibited tumor growth inhibition, including decreased tumor volumes (Fig. 7B) and tumor weights (Fig. 7C), but body weight was unaffected (Fig. 7D). Tumor growth was significantly delayed in the combination treatment group compared to the Ven group (Fig. 7B and C). The TUNEL assay results revealed that the combination treatment induced more intense DNA fragmentation and eventually led to tumor cell death (Fig. 7E), accompanied by reduced expression of Ki67, a cell proliferation marker (Fig. 7F). The Dac, Ven, and the combination treatment groups exhibited increased Cl-caspase-3 expression, and decitabine treatment increased GSDME expression (Fig. 7F). The abundance of Cl-caspase-3 and GSDME-N was further increased in the combination treatment group, as shown by western blotting (Fig. 7G). These findings indicate that treatment with HMAs in combination with Ven induces proliferation inhibition and pyroptosis in AML cells via modulation of caspase-3/GSDME activation.

## Discussion

Combination treatment with Ven and HMAs results in responses in up to 70% of patients with newly diagnosed AML, with a median time to optimal remission of 2.1 months, suggesting a unique mechanism of action for this combination therapy [35]. In this study, we found that the Bcl-2 inhibitor Ven induces pyroptosis in AML cells by activating GSDME through the intrinsic (mitochondrial) apoptotic pathway. Ven disrupts the interaction of Bcl-2 and Bax, leading to mitochondrial dysfunction, which results in the release of cytochrome C and activation of caspase-3/GSDME. Treatment with the HMA decitabine restores the expression of GSDME in AML cells, increasing their susceptibility to pyroptosis. Thus, we proposes a novel mechanism of action related to the treatment of AML with Ven and HMAs. Considering these results, the model shown in Fig. 7I was established.

Pyroptosis is a proinflammatory mode of PCD that causes the perforation of cell membranes and the release of cellular contents and is activated mainly through cleavage of GSDMs [36, 37]. Pyroptosis has a broader and more rapid tumoricidal effect than apoptosis and is a potentially novel mechanism of immunogenic cell death, similar to necroptosis and ferroptosis [38, 39]. Several main and alternative pathways of pyroptosis have been elucidated. In the main pathways, pyroptosis is induced by GSDMD, and inflammatory caspase-1/11 (classical pathway) or caspase-4/5 (nonclassical pathway) are involved [37, 40]. Among the alternative pathways, caspase-3/GSDME-mediated pyroptosis has received the most attention [23, 41]. Recent studies have shown that caspase-3 activation induced by chemotherapy or molecularly targeted drugs induces pyroptosis via specific cleavage of GSDME, resulting in growth inhibition [23, 42, 43].

Previous studies focused on the antitumor effects of Ven related to apoptosis. However, we found that Ven-treated AML cells exhibited pyroptosis characterized by cell swelling, membrane blebbing, LDH release, and GSDME cleavage. Our findings contrasted with those of previous studies where GSDME mediated the transition from apoptosis to pyroptosis but total cell death was unaltered [44], as increased GSDME expression was shown to promote Ven-induced cell death in our study. Recent studies have shown that N-GSDMs promotes rapid mitochondrial collapse and cytochrome C release through mitochondrial membrane perforation, thus activating caspase-3 [45, 46]. Our study also confirmed that the MMP signal was markedly decreased in GSDME-positive cells. This positive feedback mechanism may explain the increase in cell death upon overexpression of GSDME. Consistent with the important role of caspase-3 in pyroptosis, treatment with caspase-3 specific inhibitors was found to significantly reduce pyroptosis [23]. ROS also play an important role in pyroptosis. For example, lobaplatin induces caspase-3/GSDME-mediated pyroptosis by increasing ROS levels in colon cancer cells [30]. Iron-mediated ROS elevation promoted the oxidation of the mitochondrial outer membrane protein Tom20 in melanoma cells and induced pyroptosis by activating the Bax/Caspase-3/GSDME pathway [47]. In our study, Ven mediated Bax dissociation from Bcl-2 and increased ROS production and cytochrome C release, leading to caspase-3 activation. Caspase-3 activation and GSDME cleavage were reduced by treatment with ROS scavengers and cytochrome C release inhibitors. Thus, Ven induces caspase-3/GSDME-mediated pyroptosis via the intrinsic apoptotic pathway.

The pyroptosis effector protein GSDME is associated with several types of cancer, ncluding colon, breast, and gastric cancer, exhibiting differential expression and methylation patterns between tumor and normal tissues [32, 33, 37, 48, 49]. GSDME methylation was detected in primary gastric tumors, and exogenous expression of GSDME in cell with GSDME silencing inhibited colony formation [48]. GSDME promoter methylation was highly correlated with lymph node metastasis in breast cancer, and overexpression of GSDME in colon cancer cell lines significantly reduced the growth and colony-forming ability of these cells [32, 33]. In addition, GSDME expression is associated with good prognosis after chemotherapy in a variety of cancers and may be a potential prognostic biomarker [49]. Aberrant DNA methylation is common in AML and is associated with recurrent mutations in epigenetic regulator proteins, including IDH1/2, TET2, and DNMT3A [50]. Notably, DNMT1 promotes the self-renewal ability of leukemic stem cells and mediates resistance to conventional chemotherapy and radiotherapy[34, 51]. However, GSDME promoter methylation and expression levels have not been reported in AML. The level of GSDME promoter methylation was higher but the GSDME expression level was lower in AML patients than in normal controls, according to GSDME gene analyses and public database analysis. Treatment with HMAs reduced the GSDME promoter methylation level and increased the GSDME expression level. Low expression of GSDME was associated with poor prognosis, suggesting that GSDME may act as a tumor suppressor in AML.

Treatment with Ven combined with HMAs shows superior therapeutic efficacy in AML [19, 35, 52, 53]. Mechanistically, the combination of Ven and HMAs synergistically activates mitochondrial apoptosis in AML cells by reducing the level of Mcl-1, a potential factor in Ven resistance [54]. ROS induction is an important process promoting the cytotoxicity of several AML therapies. Decitabine increases the nuclear translocation of Nrf2 and activates the HO-1/NQO1 antioxidant pathway. In contrast, Ven disrupts this process by targeting Nrf2 for ubiquitination and proteasomal degradation [55]. Recent studies have shown that Ven directly increases T-cell function, while azacitidine induces a viral infection-like response in leukemic cells by activating the STING-cGAS pathway to induce a type I interferon response, thus increasing the sensitivity of AML cells to T- cell-mediated killing [56]. In this study, we found that the decitabine restored GSDME expression in AML cells and intensified pyroptosis in combination with Ven, suggesting a novel mechanism of combined action.

In summary, our study demonstrates that Ven induces caspase-3/GSDME-mediated pyroptosis by increasing ROS production and cytochrome C release. Genetic overexpression or demethylation of GSDME restored GSDME expression and increased Ven-induced pyroptosis in AML cells. These data provide important mechanisms and new insights to explain the clinical activity of Ven and HMAs in AML. Moreover, GSDME is downregulated in AML, and GSDME downregulation is associated with poor prognosis, suggesting that GSDME expression is a potential prognostic biomarker and that modulation of GSDME expression is a therapeutic strategy for AML.

### Supplementary Information

**Additional file 1: Figure S1.** Fluorescence microscopy detection of MMP in AML cells. **Figure S2.** GSDME expression in AML in the public database. **Figure S3.** Magnification images of primary AML cells of 2 patients after venetoclax, decitabine and combination therapy. **Figure S4.** Effect of GSDME expression on LDH release. **Table S1. **Relationship between GSDME expression andclinicopathologic characteristics in 36 AML patients. **Table S2.** The GSDME shRNA oligonucleotide sequences. **Table S3.** All RT-qPCR primers in this manuscript. **Table S4.** The information of antibody. **Table S5.** Primer for BSP (bisulfite sequencing PCR) and primer pairs specific to methylated (M) and unmethylated (U) GSDME promoter sequences. **Table S6.** Differential analysis between case and control from the human disease methylation database.

## Data Availability

All data generated or analyzed during this study are included in this manuscript.

## Abbreviations

AML: Acute myeloid leukemia
Bcl-2: B-cell lymphoma 2
HMAs: Hypomethylating agents
PCD: Programmed cell death
DNMT: DNA methyltransferases
BMMNCs: Bone marrow mononuclear cells
IHC: Immunohistochemistry staining
TUNEL: Terminal deoxynucleotidyl transferase dUTP nick end labeling
