# Supplementary material for: Antileukemic effect of venetoclax and hypomethylating agents via caspase-3/GSDME-mediated pyroptosis
Source: J Transl Med. 2023 Sep 7;21:606. doi: 10.1186/s12967-023-04481-0 (PMC10486003; doi:10.1186/s12967-023-04481-0)
Supplement: Supplementary file 1 — Additional file 1: Figure S1. Fluorescence microscopy detection of MMP in AML cells. Figure S2. GSDME expression in AML in the public database. Figure S3. Magnification images of primary AML cells of 2 patients after venetoclax, decitabine and combination therapy. Figure S4. Effect of GSDME expression on LDH release. Table S1. Relationship between GSDME expression andclinicopathologic characteristics in 36 AML patients. Table S2. The GSDME shRNA oligonucleotide sequences. Table S3. All RT-qPCR primers in this manuscript. Table S4. The information of antibody. Table S5. Primer for BSP (bisulfite sequencing PCR) and primer pairs specific to methylated (M) and unmethylated (U) GSDME promoter sequences. Table S6. Differential analysis between case and control from the human disease methylation database. [file 12967_2023_4481_MOESM1_ESM.pdf]

Supplementary Figures

Figure S1

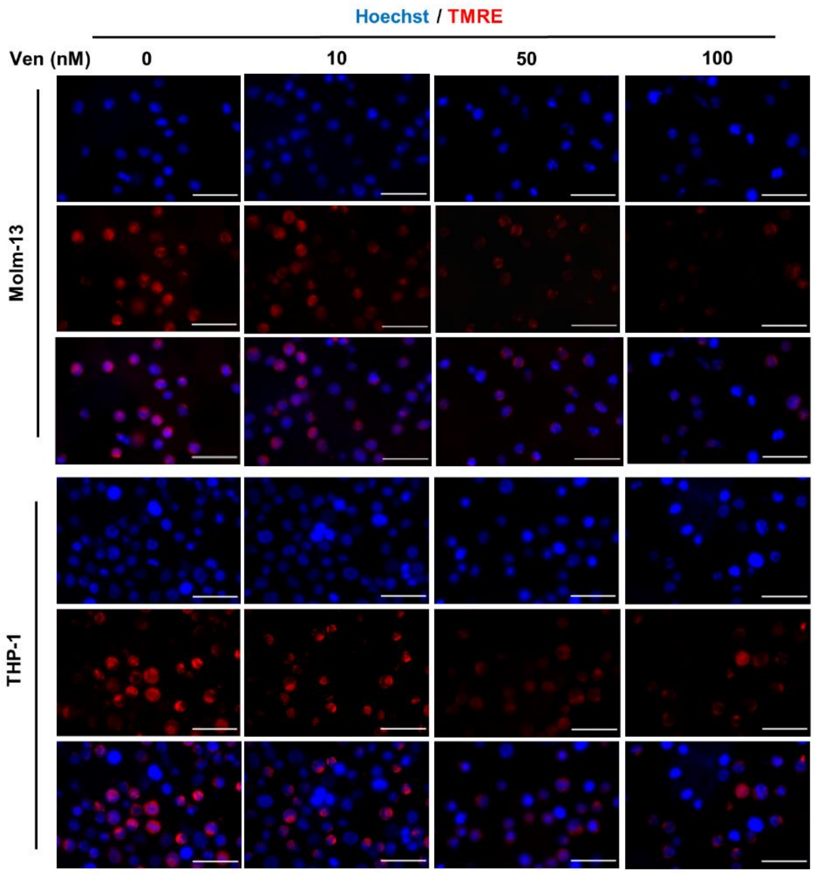

Figure S1 Fluorescence microscopy detection of MMP in AML cells. Molm-13 and THP-1 cells were treated with Ven for 24 h, followed by TMRE staining. The MMP was visualized by fluorescence microscope, scale bar: 50 μm. TMRE (red fluorescence) was used to dye mitochondria, and Hoechst 33342 (blue fluorescence) the cell nucleus.

Figure S2

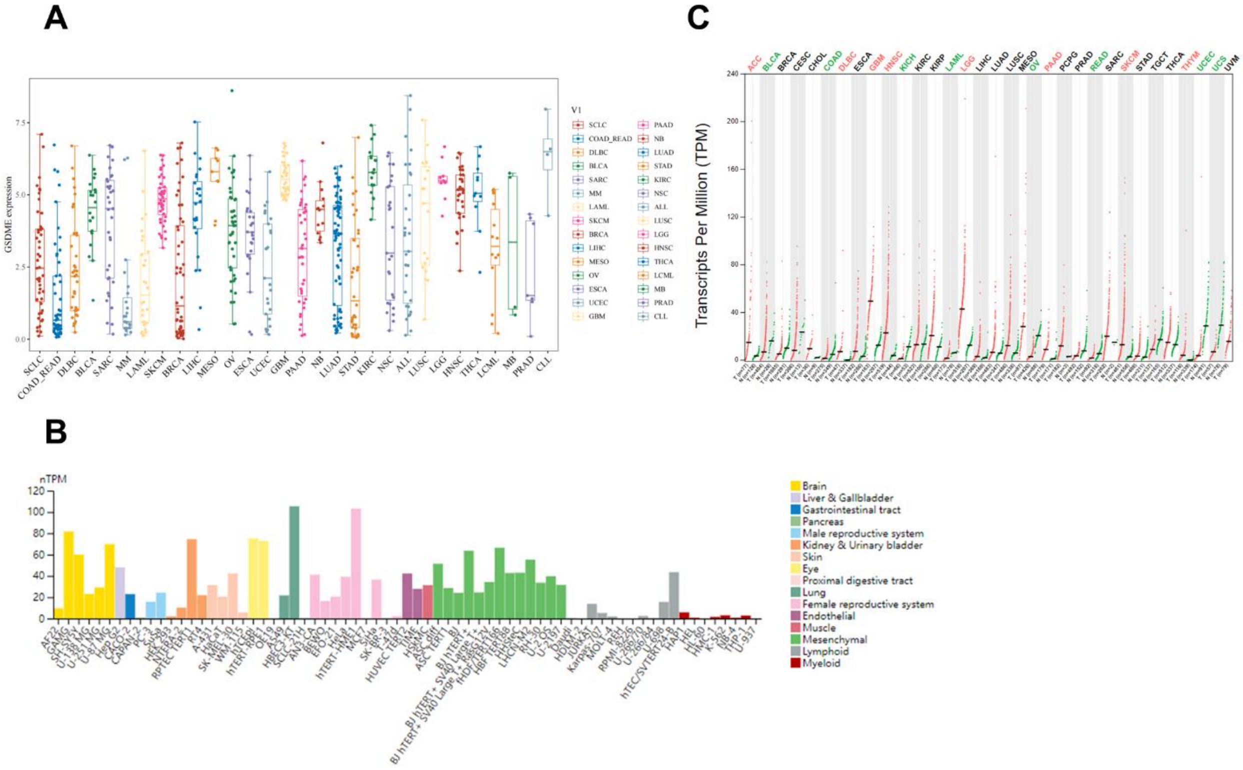

Figure S2 GSDME expression in AML in the public database. (A) The expression of GSDME in 30 types of human cancer cell line in the Cancer Cell Line Encyclopedia (CCLE) database. (B) The expression of GSDME in human cancer cell lines in Human Protein Atlas (HPA) database. (C) The expression of GSDME in 33 types compared with normal subjects in Gene Expression Profiling Interactive Analysis (GEPIA) database.

Figure S3

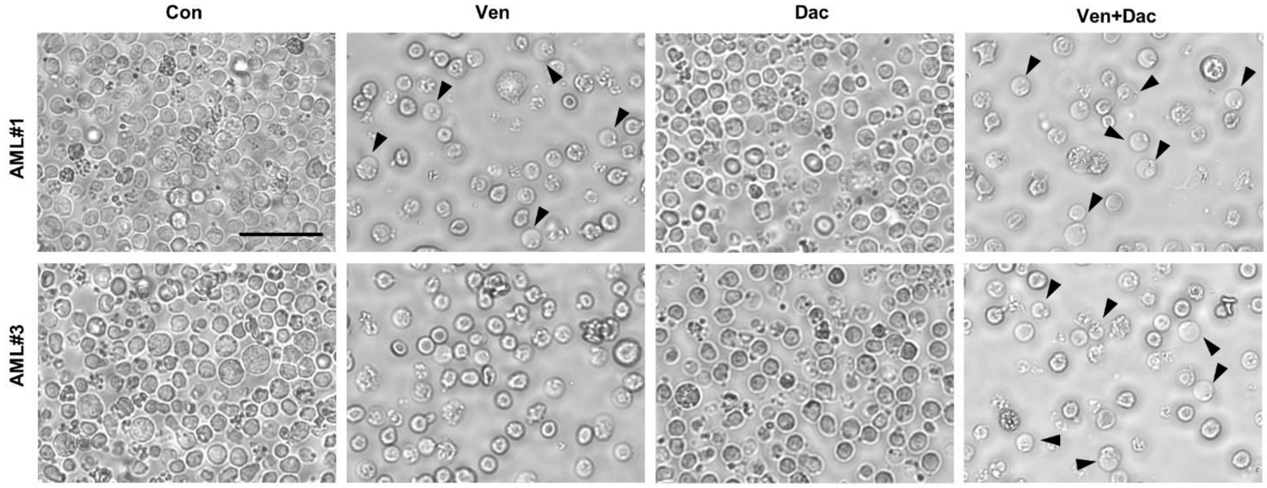

Figure S3 Magnification images of primary AML cells of 2 patients after venetoclax, decitabine and combination therapy. scale bar: 50 μm.

Figure 4

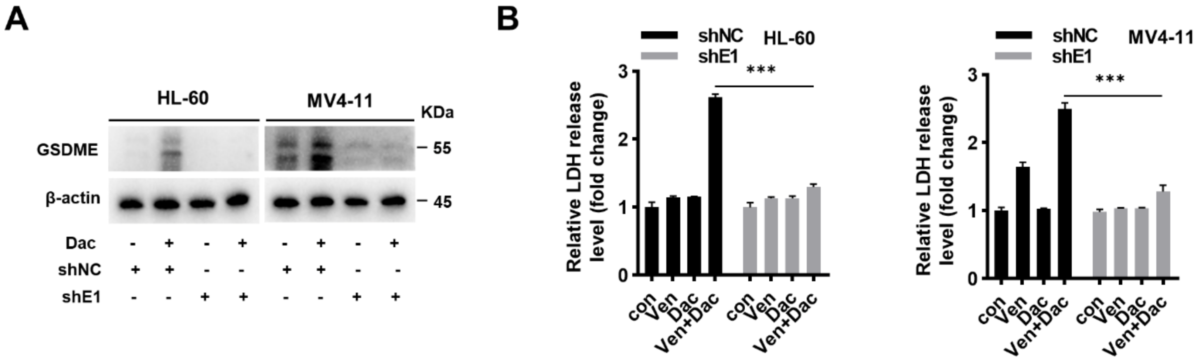

Figure S4 Effect of GSDME expression on LDH release. (A) GSDME was quantitated by western blot after different treatments. (B) Relative levels of LDH release after different treatments. β-actin was used as an internal control for western blot. $^{***}p < 0.001$.

Supplementary Tables

Table S1 Relationship between GSDME expression and clinicopathologic characteristics in 36 AML patients

Characteristics	Low expression[a], n = 18 (%)	High expression[a], n = 18 (%)	*p*-value
Sex			0.738[d]
Male	9 (50%)	8 (44.5%)	
Female	9 (50%)	10 (55.5%)	
Age			0.735[d]
<10	8 (44.5%)	7 (38.9%)	
≥10	10 (55.5%)	11 (61.1%)	
Median WBC (10^9/L, range)	23.6 (4.4-136.1)	17.65 (1-318)	0.654[c]
Median Hb (g/L, range)	82 (37-116)	81 (43-125)	0.728[c]
Median PLT (10^9/L, range)	42.5 (12-178)	48 (13-372)	0.381[c]
Median BM blasts (%, range)	54.2 (34.5-93)	56 (36-90.5)	0.792[c]
Extramedullary involvement			0.717[d]
Yes	6 (33.3%)	5 (27.8%)	
No	12 (66.7%)	13 (72.2%)	
Cytogenetics risk[b]			0.904[d]
favorable	6 (33.3%)	6 (33.3%)	
intermediate	8 (44.5%)	9 (50%)	
high	4 (22.2%)	3 (16.7%)	

a, The median ratio of the expression level of GSDME. b, Favorable risk: recurrent cytogenetic abnormalities t(8;21), t(15;17) and inv(16). Intermediate risk: normal and non-complex karyotypes. High risk: complex karyotypes. c, one-way ANOVA analysis. d, chi-square test.

Table S2 The GSDME shRNA oligonucleotide sequences

GSDME shRNA	Oligonucleotide sequences (5' – 3')
GSDME shRNA1	CCGGGCATGATGAATGACCTGACTTCTCGAGAAGTCAGGTCATTCATCATGCTTTTG
GSDME shRNA2	CCGGGCGGTCCTATTTGATGATGAACTCGAGTTCATCATCAAATAGGACCGCTTTTTG

Table S3 All RT-qPCR primers in this manuscript

Gene	Primers (5' – 3')
GSDME-F	TGCCTACGGTGTCATTGAGTT
GSDME-R	TCTGGCATGTCTATGAATGCAAA
β-actin-F	ATTGCCGACAGGATGCAGAA
β-actin-R	ACATCTGCTGGAAGGTGGACAG

Table S4 The information of antibody

Antibodies	Supplier	Cat.Number	Working Concentration
GSDME	Abcam	ab215191	WB (1：1000); IHC (1：100)
GSDMB	Abcam	ab215729	WB (1：1000)
GSDMA	Abcam	ab230768	WB (1：1000)
GSDMC	Abcam	ab225635	WB (1：1000)
GSDMD	Abcam	ab219800	WB (1：1000)
Active + pro Caspase-3	ABclonal	A19654	WB (1：1000)
Active Caspase-3 (CL-CASP3)	ABclonal	A11021	WB (1：1000); IHC (1：100)
β-actin	ABclonal	AC026	WB (1：5000)
β-tubulin	ABclonal	AC008	WB (1：1000)
Bcl-2	ABclonal	A19693	WB (1：1000)
Bax	ABclonal	A20227	WB (1：1000)
Cytochrome C	ZENBIO	R22867	WB (1：1000)
COX IV	ZENBIO	T200147	WB (1：1000)
DNMT1	ZENBIO	R24130	WB (1：1000)
HRP Goat Anti-Rabbit IgG (H+L)	ABclonal	AS014	WB (1：5000)
HRP Goat Anti-Mouse IgG (H+L)	ABclonal	AS003	WB (1：5000)
Ki67	ABclonal	A20018	IHC (1：200)

Table S5 Primer for BSP (bisulfite sequencing PCR) and primer pairs specific to methylated (M) and unmethylated (U) GSDME promoter sequences

Primer name	Primers (5' – 3')	Length	Location
H-GSDME-BSP-F	GTTTTTATAGGTTGGATAGGTTTTGG	324	1930-2253
H-GSDME-BSP-R	ACCAAACCTCCCAAAAACCC		
H-GSDME (M) - F	TTTTTGGTTGAATCGTGAGGTC	148	1959-2106
H-GSDME (M) - R	AAATAAACTACGCCTACCTTCCG		
H-GSDME (U) - F	TTTTTTTGGTTGAATTGTGAGGTT	154	1957-2110
H-GSDME (U) - R	AAAAAAATAAACTACACCTACCTTCCA		

Table S6 Differential analysis between case and control from the human disease methylation database.

Disease name	Genomic region	Transcript	Gene	*p*-value
Acute Myeloid Leukemia [LAML]	chr7:24796583-24799083	# 1 (NM_001127453)	DFNA5 (GSDME)	2.366e-12
Acute Myeloid Leukemia [LAML]	chr7:24797139-24799639	# 2 (NM_001127454)	DFNA5 (GSDME)	1.784e-09
Acute Myeloid Leukemia [LAML]	chr7:24797139-24799639	# 3 (NM_004403)	DFNA5 (GSDME)	1.784e-09
